# Seabird bycatch mitigation trials in artisanal demersal longliners of the Western Mediterranean

**DOI:** 10.1371/journal.pone.0196731

**Published:** 2018-05-09

**Authors:** Verónica Cortés, Jacob González-Solís

**Affiliations:** 1 Institut de Recerca de la Biodiversitat (IRBio), Universitat de Barcelona, Barcelona, Spain; 2 Departament de Biologia Evolutiva, Ecologia i Ciències Ambientals, Facultat de Biologia, Universitat de Barcelona, Barcelona, Spain; Hellenic Centre for Marine Research, GREECE

## Abstract

High numbers of seabirds are killed annually worldwide in longline fisheries. In the Mediterranean, this mortality is seriously affecting the viability of seabird populations, in particular of the three endemic shearwaters. Even so, there is currently no specific seabird mitigation requirements for the longline fleet operating in this area. From 2013 to 2014, we assessed the efficiency and practical applicability of four mitigation measures on artisanal demersal longliners targeting European hake (*Merluccius merluccius*) in the western Mediterranean: night setting, tori line, weighted lines and artificial baits. We performed fifty-two pairs of experimental (with the tested mitigation measure) and control settings (without any measure), and compared their effects on seabird interactions and fish catches. In addition, we estimated the longline sink rates and the seabird access area to baited hooks in different longline configurations. Night setting reduced bycatch risk without affecting target and non-commercial fish catches. The tori line may have reduced the bycatch risk by displacing bait attacks beyond the end of the line, but at this distance shearwaters could still access to the baits and the streamers did not deter birds under calm wind conditions. Weighted lines increased sink rate, but it resulted in only a minor reduction of the seabird access window to baited hooks and led to some operational problems during the setting. Artificial baits substantially reduced commercial catches. Moreover, the seabird access to the baited hooks was influenced by the longline configuration, the setting speed and the relative position to the floats and weights. So far, night setting stands out as the best mitigation measure for reducing bycatch levels without compromising target catches in demersal longliners. Ideally, these results should be confirmed in longliners targeting species other than European hake.

## Introduction

Longline fisheries constitute the most serious threat faced by seabirds at sea worldwide [1]. At least 160,000 seabirds are killed through getting hooked and drowning each year when birds attack baited hooks during line setting [2]. Procellariformes, such as albatrosses, petrels and shearwaters appear to be the most affected seabirds by this fishery [2,3]. Combined with other human-mediated disturbances, mortality induced by longline fisheries is putting numerous seabird populations at risk and has driven some to extinction [4–6]. Nevertheless, several studies have demonstrated the efficiency of different mitigation methods to reduce seabird bycatch in longline fisheries [3,7]. Possible measures include deterring birds from taking baits (e.g. tori lines, acoustic or olfactory deterrents), limiting access to the baited hooks by increasing the sink rate of the longline (e.g. weighted lines, Chilean system, thawing the bait), avoiding the periods or areas where seabird interactions are most intense and likely (e.g. night setting, area or seasonal closures) and making bait less attractive or visible for seabirds (e.g. artificial baits, blue-dyed baits) [3,7]. Nevertheless, the efficiency of the mitigation methods employed may vary depending on the seabird species assemblage at fishing grounds and the longline type used [7]. In principle, the use of mitigation methods increases fishing efficiency and profitability, since the reduction of the bait loss by seabirds could lead to an increase of commercial catches [8]. However, confirming this has remained elusive. For this reason, their effects on target species and on the fishing operations must be carefully examined.

In Western Mediterranean, bycatch by longlines is thought to be one of the main causes of population decline in shearwater species [9–11]. Bycatch mortality is significantly reducing adult survival and affecting both sexes unequally, which might be exacerbating bycatch impact on population viability [12]. Scopoli’s shearwater (*Calonectris diomedea*) is the most bycaught species in this region [13–15], as it was also observed in central and Eastern Mediterranean [16,17]. Nevertheless, a previous study on bycatch rates showed that Balearic and Mediterranean shearwaters (*Puffinus mauretanicus* and *P*. *yelkouan*) are also frequently caught by demersal longliners [18]. The current situation of the critically endangered Balearic shearwater is particularly alarming, as a recent review on its demographic trends highlighted again the unusually low adult survival, which is leading to a severe decline of its populations [9]. In fact, this study showed Balearic shearwaters could be driven to extinction in 60 years if mortality caused by fisheries continues at its current level.

Artisanal demersal longliners seem to be the major source of seabird mortality in the Mediterranean sea [19]. This fleet uses smaller bait and hooks compared to pelagic longliners thus increasing the chances of birds being hooked. Massive catches of tens to hundreds shearwaters in only one setting occur occasionally every year in this fishery suggesting that demersal longliners are having a large effect on the three endemic shearwaters of the Mediterranean basin [18]. The Balearic and the Mediterranean shearwater are the most affected species in these large scale mortalities, mainly due to their gregarious behaviour and diving abilities, allowing them to seize baited hooks at greater depths than other seabirds such as gulls [18]. Despite the potential impact that demersal longliners are having on shearwater populations, no specific mitigation method is currently required by fishery management authorities, or used to minimize seabird bycatch. Therefore, there is an urgent need to find (and implement) effective mitigation techniques adapted to the Mediterranean demersal longline fleet.

Seabird bycatch by the demersal longliners operating in the study area increases during the breeding period and when longlines are set at sunrise [13,18]. In addition, some operational characteristics of the vessels also have a significant influence, such as the bait type used, gear configuration (in particular, the distance between the weights) and the number of hooks set [18]. In accordance with these findings, we selected four mitigation methods, which have proved to be effective in other regions, and adapted them to the artisanal demersal longliners from the Western Mediterranean: (1) night setting, (2) tori lines (bird scaring lines or streamer lines), (3) weighted lines, and (4) artificial baits. Then, we tested their efficacy and practical applicability. For each method, we assessed their effects on seabird bait attacks, on fish catches and on fishing activity by comparing paired longline settings with and without each mitigation method [7]. In addition, we determined the sink rates of the longline configurations most commonly used in demersal longliners of the study area.

## Methods

### Ethics statement

The mitigation trials were conducted in two commercial vessels, so all fishing activities were performed by the fishermen. We had the approval of the captains to carry out the experiments in their vessels and complied with the relevant requirements to go onboard commercial fishing vessels as an occasional crewmember. This study did not require any special permission since fishing operations were conducted in strict conformity with the local rules and regulations.

### Mitigation measure trials

**Fig 1:**
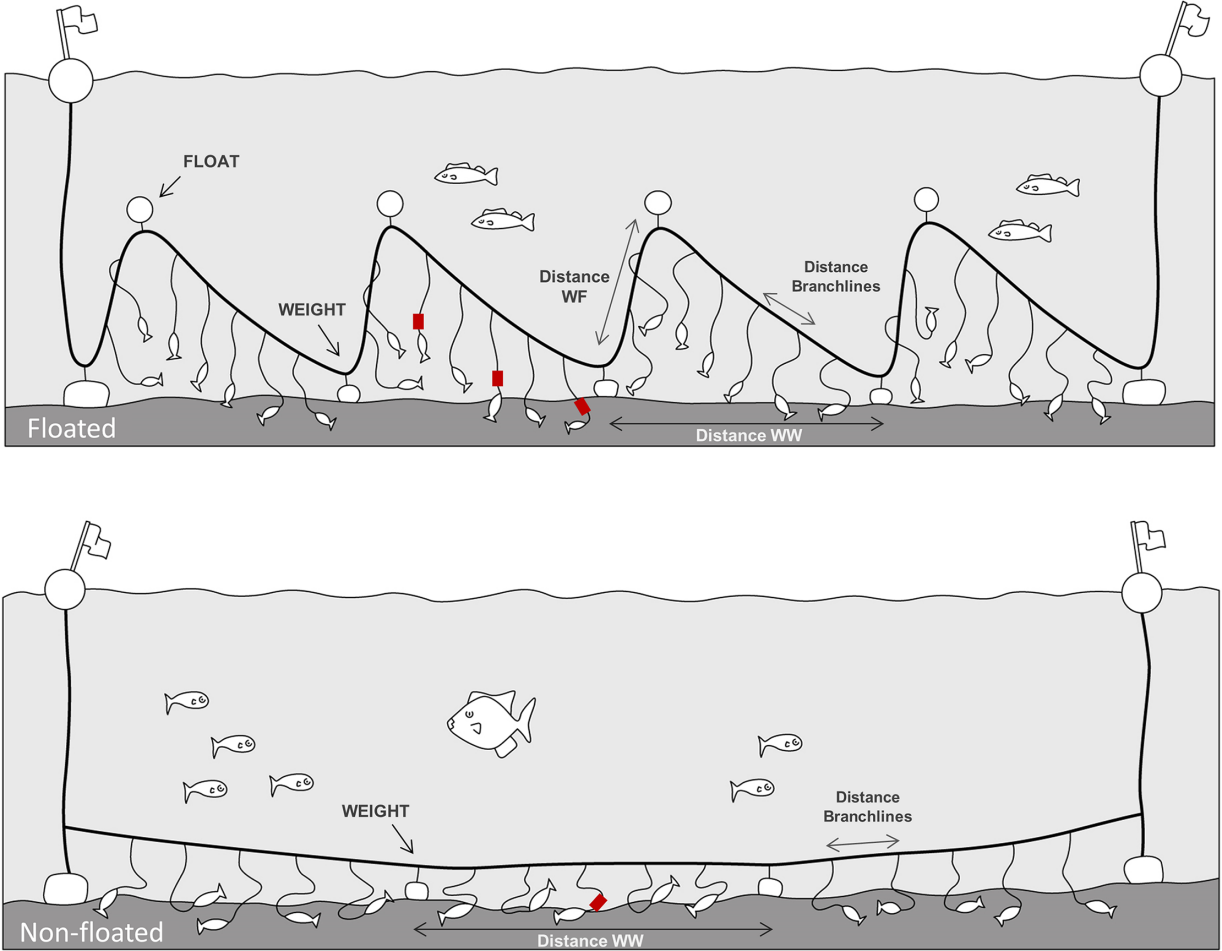
Longline types used by the artisanal demersal fleet. Floated: “Piedra–Bola” configuration composed by a sequence of floats and weights. Non-floated: fishing gear typically used by small-scale vessel where only weights are attached to the mainline. The position of the time-depth recorders (TDRs) is also indicated by a red square in the floated (Float, Mid and Weight positions) and non-floated (Mid) longlines. Drawing by Toni Mulet.

The experiments were mainly carried out on a medium-scale artisanal demersal longliner (*Cona C*.*B*., 11.10 m length) from north-western Mediterranean between May to July 2013 and 2014 (41°08’N, 1°53’E, Vilanova i la Geltrú, Catalonia, Spain). In addition, one of the trials (tori line) was also tested on another medium-scale demersal longliner in May 2014 (*Mar endins*, 14.5 m length) that operates in the Gulf of Lion area (42°20’N, 31°30’E, Llançà, Catalonia, Spain). The areas and period were chosen to be among those with the highest risk of bycatch within the study area [18]. The fishing grounds are located within important foraging areas of the most susceptible species (the three endemic shearwaters) [20–22], while the study period overlapped with the prelaying and incubation stage of Scopoli’s shearwaters and the chick-rearing and post-nuptial migration stage of the *Puffinus* shearwaters. In addition, trials performed with one of the longliners (*Cona C*.*B*.) coincided with a trawling moratorium period, which should exacerbate bycatch probability [15,23]. Both vessels used a fishing gear configuration called the “Piedra—Bola” system (Floated), which is composed of a series of floats and weights to keep hooks at different heights from seabed (Fig 1), following a zigzag structure (see [18]). They also used the same size of hook: 3.17 and 1.40 cm of total length and of gape, respectively.

Hooks were baited with European pilchard (*Sardina pilchardus)* or European anchovy (*Engraulis encrasicolus*) to target European hake, although other species with less commercial value were also caught, such as blackmouth catshark (*Galeus melastomus*), European conger (*Conger conger*), blackbelly rosefish (*Helicolenus dactylopterus*) and small-spotted catshark (*Scyliorhinus canicula*). There were some differences in the fishing gear configuration linked to the particular practices of the fishermen on the different boats (Table 1). The main differences were the distance between consecutive weights, as well as the distance from the weight to the float.

**Table 1:** Characteristics of the fishing gears used in demersal longliners in which the sink rates were recorded.

	Weights (kg)	Distance WW (m)	Distance WF (m)	Distance Branchlines (m)	Branchline length (m)
Floated 1	2.3	70	11	3.7	1.8
Floated 2	2.3	90	7	3.3	1.8
Non-floated	1.5	500	-	8	3

Longline types: “Piedra–Bola” configuration used by the medium-scale vessels *Cona C*.*B*. (Floated 1) and *Mar endins* (Floated 2), and the small-scale vessel *Vigilant* (Non-floated). Distance WW = distance between consecutive weights. Distance WF = minimum distance between the weight and the float.

Trials were performed under typical fishing conditions to test different mitigation strategies. A description of the methods used is detailed in the Supporting information (S1 Table). For each mitigation measure, paired longline settings were performed consecutively: one line was normally set without any mitigation measure (control setting) and the other one using the tested mitigation measure (experimental setting). In case of night setting trials, the night set was scheduled to be completed 1 hour before the sunrise (when nautical dawn begins: sun angle is 12° below horizon), while the day set was performed between 1 and 3 hours after the night set, coinciding with the sunrise or the first hours of the day. The duration of each setting (control or experimental) was about 30 minutes. In case of the *Cona C*.*B*., longlines were left in the water until the night of the following day (around 15 hours), starting with the first line set the previous day. On the *Mar endins*, the soak time of the longlines was 2–3 hours and the order of the hauling operations was the same as for the settings.

The artificial baits used in the trials were manufactured by the Spanish company Arom Bait^®^, and consisted of brown, triangular pieces composed of a mix of products derived from fish.

During setting operations, the number and species of seabirds attending the vessel and the attacks on bait performed by each species every 10-min period were recorded. For each time interval, the number and distance astern of overall attacks on bait were also registered and were ranked in six intervals: < 5, 5–10, 11–20, 21–50, 51–100 and > 100 m. During the night, counts of birds and attacks were limited by the distance from the stern at which these could be detected given the illumination provided by the boat (< 50 m). Additionally, a 12-volt halogen spotlight was used to confirm seabird species and increase the detection of bait attacks during the night settings. Position, depth and setting speed were also recorded at every count interval. A detailed description of the fishing gear used and the meteorological data were registered for each fishing trip. Seabird catches were recorded during the hauling, together with the number of the fish commercial catches, their size (from the tip of the snout to the tip of the longer lobe of the caudal fin), condition and the discards (fish catches returned to the sea). Seabird catches observed during the setting but not retrieved during the hauling were also considered. Discards were classified into four different causes: (1) bad condition of the specimens, (2) low commercial value, (3) small size and (4) non-commercial or protected species (fish bycatch). Moreover, the body mass of a sample of 470 European hakes were measured to obtain the relationship between the weight and the size, and then estimate the body mass from those individuals that were not weighed. Potential function showed a best fit of these data (R^2^ = 0.958; p < 0.005):

$$Body\;mass=0.012*{Total\;length}^{2.899}$$

The effectiveness of each mitigation method in reducing seabird attraction and bait attacks was assessed comparing results between the control and experimental lines. The practical applicability was also evaluated for all mitigation measures tested, while the effects on fish catches was only assessed in those measures that involved a change in the longline configuration and fishing habits (night setting, weighted lines and artificial baits). Given that seabird incidental catches only occur occasionally (12% of the fishing days, [18]), it was not possible to obtain enough data to compare bycatch rates between control and experimental settings. Instead, the bait attacks were used to infer seabird bycatch risk [18].

### Determining longline sink rate

Sink rates were measured to 10 m depth with Time-Depth Recorders (TDRs, long-life G5 data storage tags, CEFAS technology limited, UK). This reference depth was based on the dive ability of the Balearic shearwater [22]. Each TDR measured 35.5 mm length and weighed 2.5 g in seawater. TDRs were programmed to record depth within 0.5 m every second. Devices were attached onto the monofilament of the branchline at 5–10 cm from the hook.

Sink rates were estimated for the three different longline configurations used in demersal longline vessels of the study area (Table 1 and Fig 1). Most data were collected from the control longlines (Floated configurations) of the mitigation trials conducted on the *Cona C*.*B*. (Floated 1) and *Mar endins* (Floated 2). TDRs were also deployed on a small-scale vessel called *Vigilante* (6.81 m length) from Mallorca (Balearic Islands) that used a different longline configuration (Non-floated). In this case, the longline only had weights attached at regular intervals, which keeps the hooks level over the seafloor (Fig 1). Moreover, in contrast to other configurations studied, the weights used by this vessel were less heavy and more widely spaced (Table 1). This longline type is typical in the polyvalent small-scale vessels from the western Mediterranean to target a wide variety of demersal fishes, such as common pandora (*Pagellus erythrinus*), toothed bream (*Dentex dentex*) or gilt-head bream (*Sparus aurata*) (see [18]).

TDRs were placed in different sections of the longline: initial (first 50 hooks), middle and final (last 50 hooks). Within each section, TDRs were placed at different hooks inside the float-weight sequence (see Fig 1): near to the float (Float), between the float and the weight (Mid) and near to the weight (Weight). In the case of the non-floated longlines, the TDRs were located between consecutive weights (Mid). During the trials of the additional lead weights, TDRs were deployed in the two-paired longlines of each sample (control and experimental). To check if the lead weights increased the sink rate of the hooks, the devices were placed at the same section of the longline and position inside the float-weight sequence.

From the sink rate data and the setting speed of the vessel, it was possible to determine the distance behind the vessel at which shearwaters are most vulnerable to becoming hooked, namely the seabird access window (vessel speed x seconds to 10 m depth). This allowed the greatest bycatch risk area behind the stern during the setting for each longline type to be defined.

### Tori line

**Fig 2:**
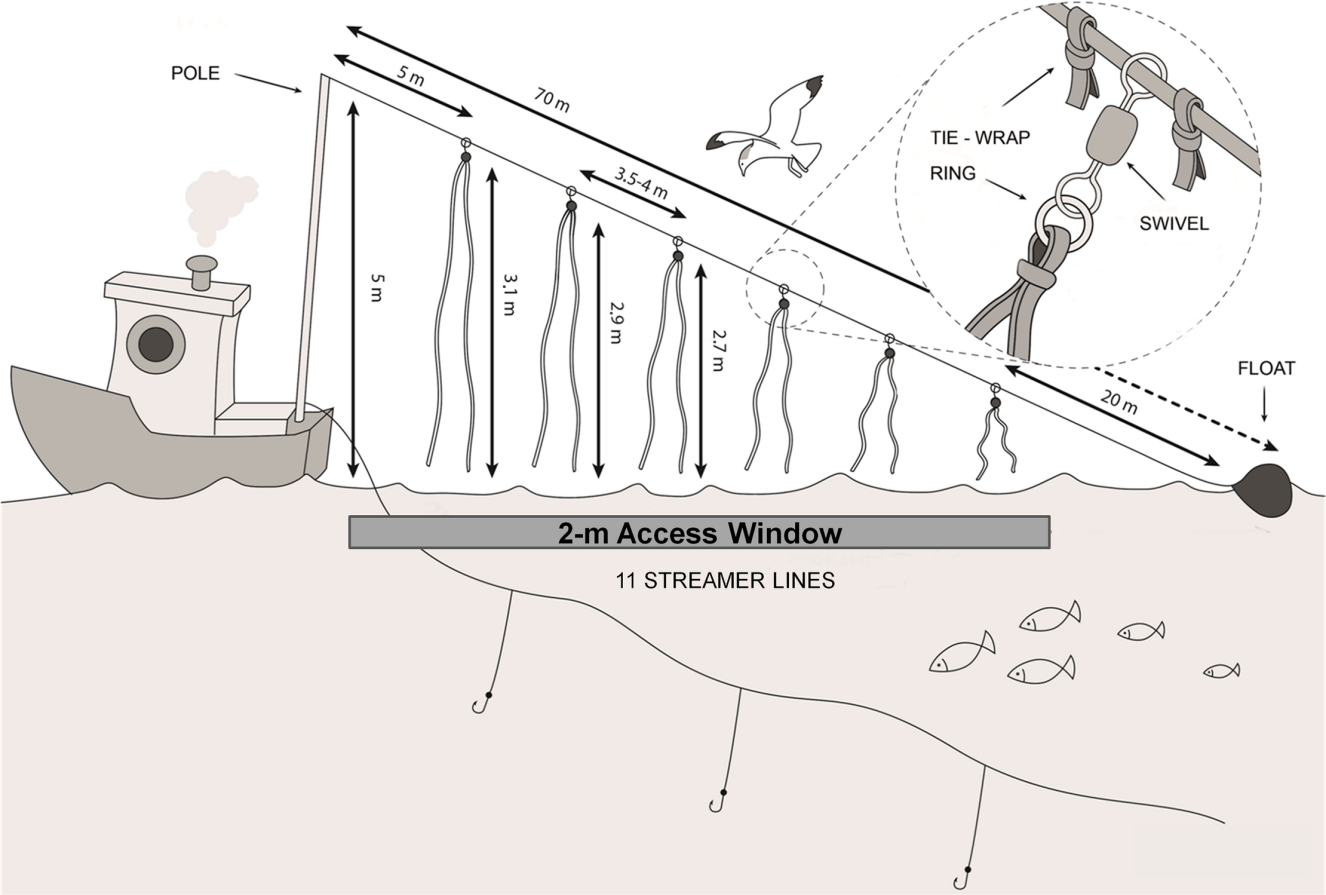
Design of the tori line used in the trials. Drawing by Toni Mulet.

The tori line was designed following the guidelines and recommendations for demersal longline vessels [24,25], and it was then adapted to the traits and size of local vessels with the help of the fishermen. Before the experiments, the height of the tori line, length and distance between streamers were modified until adequate protection of the baited hooks was achieved. The aerial extent of the streamers determines the efficiency in deterring birds from the sinking baited hooks [24], so the tori line was set as high as possible to reach sufficient aerial extent to cover as much as possible of the seabird access window. The final configuration of the tori line (Fig 2) was composed of 11 paired branch streamers distributed along a 6 mm-wide twisted rope of 70 m length. Rope was fixed on a pole located at the stern 5 m above the water surface. Brightly coloured long streamers were used to scare birds from flying under the aerial extent of the tori line [24,26]. Each streamer was 12.5 mm thermoplastic red tubing, decreasing in length with increasing distance from the vessel (3 m to 1 m long) and attached to the rope at intervals of 4 m. All streamers reached the sea-surface in calm conditions. A float was added at the end to create drag and tension of the rope. This kept the line off the water for some distance and, consequently, increased the aerial extent of the streamers. The main purpose was to cover a minimum of 20 m behind the vessel, as most seabird attacks (89%) in demersal longliners operating in the area occur within 5–20 m behind the vessel [18]. In each sample, consecutive settings with or without the tori lines deployed were conducted in the same order (first set without the tori line) to compare its effect on seabird interaction (details in Supporting information). Moreover, during the trials, additional tori line settings were also performed to better assess its adaptation to the vessels.

### Statistical analysis

Generalized linear mixed models (GLMMs) were used to test the effect of the mitigation measure on the number of bait attacks, the number and total kilograms caught of hake, the number of the most common commercial non-target species (catsharks and congers) and the proportion of fish discarded. The seabird species not vulnerable to bycatch, such as the Mediterranean storm-petrel (*Hydrobates pelagicus melitensis*), individuals discarded of target species and the samples of the fish catches data in which the paired lines differed more than 20 m depth were not considered in the analyses. Each model included the use of the mitigation measure as a binary fixed effect. “Trip” was included as a random term to correlate the observations of paired longline settings. When the number of hooks differed between paired longline settings, this variable was considered as an additional factor (seabird observations in tori line trials) or offset (fish catches in weighted lines). The number of birds following the boat was used as an offset [27,28], except in the case of the night-setting trials due to the difficulty in detecting birds in low-light conditions. Moreover, in this latter case, the effect of the measure on seabird interactions was assessed considering only the attacks occurred in the first 50 m from the stern, that is the distance in which the attacks could be detected during the night by the illumination of the boat. Therefore, the number of birds that attacked baits at this distance is unknown as these counts were not divided by distance intervals as it was done for the bait attacks. For GLMMs, significance tests for fixed effects were performed using Wald *Z*-tests [29]. Models were fitted with the laplace approximation using the package “glmmADMB”. A negative binomial distribution was assumed for the response variable of the seabird observations, given that count data typically show a skewed distribution and overdispersion [30,31]. For the fish catches, the distribution of the response variable of each dataset was previously checked for normality using Shapiro-Wilk test. In case of no deviation from normality, linear mixed models (LMMs) were applied by using the “lmer” function from the “lme4” package. Then, a likelihood-ratio test (LT) was performed to obtain the p-values. Otherwise, negative binomial GLMMs were used in accordance with the type of mean–variance relationship found in the response variable [31]. On the other hand, a gamma distribution was used for the kilograms of hake and a binomial distribution for the proportion of fish discarded [31]. In case of the congers, due to their relative lower abundance, the probability of catching the species was modelled using binary data with a Bernoulli distribution.

The effect of the measure on the type of fish discarded was also assessed by dividing the discards into different reasons (fish damaged, low value, small size, bycatch) and comparing their proportion between control and experimental settings. Moreover, the effect of the tori line on the distance astern where the bait attacks occurred was evaluated comparing the proportion of attacks inside (< 50 m) and outside (> 50 m) the area covered by the streamers. These two last analyses were assessed using a chi-square test.

Sink rate differences among (1) longline configurations, (2) the positions along the float-weight sequence and (3) the lines with and without additional lead weights, as well as the differences in the extent of the seabird access window between longline vessels, were compared using an analysis of variance (ANOVA) and a post–hoc analysis (Tukey’s test). P-values were corrected by Bonferroni method. To compare sink rates between sections a Welch's *t*-test was used. Differences between longline configurations were evaluated by considering the values obtained in the middle sections and between consecutive weights (Mid positions). However, only the sink rates obtained in the Floated 1 configuration were used to assess the differences between the sections and the positions along the mainline. Values obtained in the Mid position were used to compare sections, while in the case of the comparison between hook positions in the float-weight sequence the data from the initial section was used. The data from the final section and Weight sink speed values from the middle section were not considered because of their small sample size. On the other hand, to assess the effects of lead weights on hook sinking speed, the sink rates recorded on hooks located in the middle section and near to the floats (Float) were considered. All statistical analyses were conducted using R version 3.2.4.

## Results

### Bait attacks

**Fig 3:**
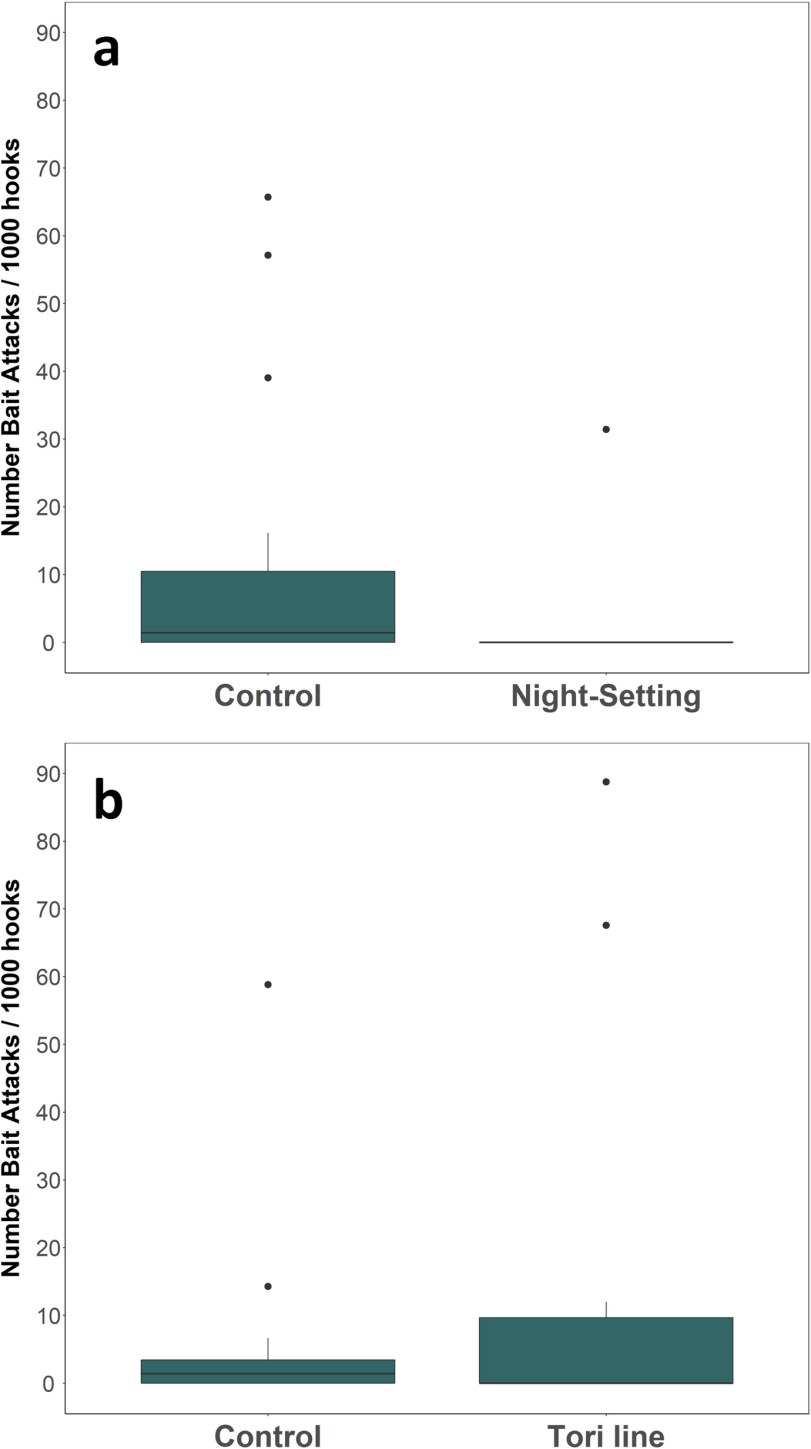
**Number of bait attacks in the night setting and tori line trials (a and b).** Attacks are shown per 1000 hooks set. In each trial, paired longlines were set; “Control” denotes the settings without mitigation measure. The middle line of boxes shows the median and the filled circles represent outliers.

Setting at night was the only measure that significantly reduced the number of bait attacks (see Table 2 and Fig 3, Negative binomial GLMM: coefficient = -4.87, SE = 1.51, Z = -3.23, p <0.001). Unfortunately, only a small numbers of bait attacks were registered in the trials of the additional lead weights and artificial baits, so it was not possible to assess their effects on seabird interactions. This is because bait attacks occur in relatively low frequency in the demersal longlines of the study area (38% of the sets, [18]) and, in addition, both trials were performed at the end of the peak season of bycatch.

**Table 2:** Bait attacks registered by species (those most susceptible to bycatch) and mitigation measure.

Species	Control	n	Night setting	n	Control	n	Tori line	n
*Calonectris diomedea*	7.33 (0.95–15.19)	6	1.57 (0–4.71)	1	5.49 (0.49–14.7)	7	13.16 (0.63–30.58)	4
*Puffinus mauretanicus*	0.95 (0.05–2.57)	4	0	0	0.79 (0–2.30)	2	0.95 (0–2.86)	1
*Puffinus yelkouan*	0.05 (0–0.14)	1	0	0	0.75 (0–1.78)	2	0.54 (0–1.30)	2
*Larus michahellis*	0	0	0	0	0.38 (0–1.15)	1	0.69 (0–2.08)	1
*Larus audouinii*	2.62 (0.95–4.67)	8	0	0	0.64 (0–1.59)	2	0	0
*Total*	11.00 (3.52–20.47)	11	1.57 (0–4.71)**	1	6.49 (1.05–15.40)	8	13.15 (1.19–29.15)	5

*** *p< 0*.*001*

** *p < 0*.*005*

* *p < 0*.*05*

Number of bait attacks (mean and 95% confidence intervals) per 1000 hooks of the most common seabirds interacting with longline vessels for each mitigation measure: night setting (N = 20 paired sets) and tori line (N = 12). "Control" refers to settings without mitigation measure. n = number of samples in which an interaction event occurred. “Total” refers to the bait attacks performed by all species interacting with vessels. Confidence intervals (95%) were determined using bootstrap re-sampling (10000 iterations) from observed data.

During night settings, seabirds following the vessels were those that are known to have nocturnal foraging habits (Table 2), such as Scopoli’s shearwaters [32] and Audouin’s gulls (*Larus audouinii*) [33]. Nevertheless, only Scopoli’s shearwater was seen to attack bait in only one of the samples performed during the new moon.

**Fig 4:**
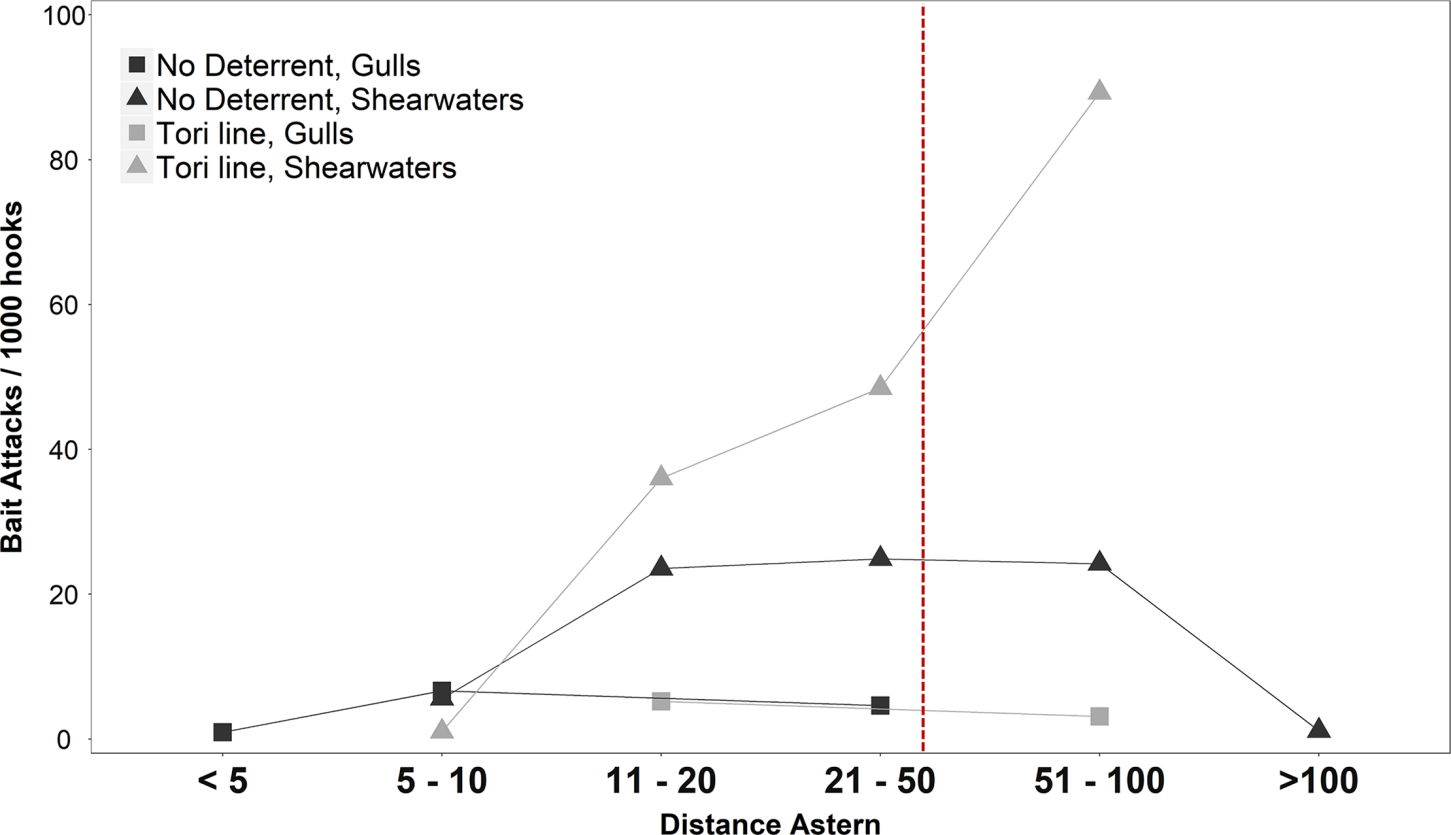
Total number of attacks registered at different distance intervals from the vessel stern. Shearwaters (triangle; Scopoli’s, Balearic and Mediterranean shearwater) and gulls (square; Audouin’s and yellow-legged gulls) were considered separately. Dark refers to the control settings without tori line (No deterrent, n = 12 settings), while grey refers to experimental settings with the tori line installed (Tori line, n = 12). Dashed line denotes the limit of the area covered by the streamers.

The number of bait attacks did not differ between control and tori line settings (Fig 3, Negative binomial GLMM: coefficient = 0.19, SE = 0.51, Z = 0.37, p = 0.71). Nonetheless, the tori line increased the distance at which bait attacks occurred (Fig 4), scaring seabirds away from the area covered by the streamers lines (> 50 m; χ^2^ = 14.56, *df* = 1, *p* < 0.005). This was particularly true for shearwaters (Fig 4), which continued to attack baits when the tori line was deployed but just further away from the stern. However, the attacks on bait also occurred inside the tori line area (< 50 m, 48% of the attacks observed).

During the trials, three Mediterranean shearwaters were caught in one of the samples; one of them in a control setting and the other two in a tori line setting, and one Scopoli’s shearwater was caught in another sample during the tori line setting. On the same day, three birds were caught (one Scopoli’s shearwater, one Mediterranean shearwaters and one Balearic shearwater) during an additional tori line setting performed with out the paired comparison settings. All seabird catches occurred on a calm day. Bait attacks occurred with the same frequency inside of the area covered by the streamers in calm (< 50m, 51%, N = 102 attacks) and windy days (45%, N = 76). However, these were proportionally greater in the first 20 m from the stern on calm days (< 20 m; χ^2^ = 5.43, *df* = 1, *p* < 0.05). In addition, attacks near the stern were also favoured when fishermen stopped the boat for changing the box where longlines are stored (each box contains at about 420 hooks). During this time lapse, the aerial extension of the tori line was reduced considerably, allowing birds to attack baits closer to the boat.

We observed that the aerial coverage of the streamer lines varied with weather conditions and setting speed, but it usually covered 30–45 m astern. Taking into account the mean setting speed and sink rate, the streamers covered the sinking hooks until they reached 2 m depth below the surface.

### Commercial catches

Target catches did not differ between control and night settings or lead weight settings (Table 3). However, we found a significant increase of the Blackmouth catshark in longlines with additional weights (Negative binomial GLMM: coefficient = 0.75, SE = 0.34, Z = 2.19, p < 0.05) and higher likelihood of catching European congers when longlines were set at night (Bernoulli GLMM: coefficient = 18.75, SE = 5.52, Z = 3.40, p < 0.001). On the contrary, we found a significant reduction in the number (Negative Binomial GLMM: coefficient = -1.49, SE = 0.19, Z = -7.83, p < 0.001) and total weight caught (Gamma GLMM: coefficient = -1.54, SE = 0.20, Z = -7.69, p < 0.001) of the European hake (target species) during the artificial bait setting. Therefore, the catches obtained in artificial baits represented only a quarter of the total hake caught when fishermen used sardine or anchovy bait.

**Table 3:** Fish catches registered for those mitigation measures that involved a change in the longline configuration and fishing practices.

		Control	Night setting	Control	Weighted lines	Control	Artificial baits
***Merluccius merluccius***	***a***	35.77 ± 5.97	33.44 ± 6.00	28.89 ± 3.84	28.98 ± 4.64	57.14 ± 5.76	12.95 ± 3.22***
	***b***	20.26 ± 3.37	18.41 ± 5.59	14.56 ± 2.34	15.11 ± 2.61	35.49 ± 8.14	7.67 ± 1.71***
***Galeus melastomus***		14.01 ± 6.34	14.82 ± 5.53	36.02 ± 9.85	76.14 ± 14.71*	5.90 ± 5.67	3.05 ± 3.05
***Conger conger***		0.80 ± 0.26	1.54 ± 0.30**	1.77 ± 0.58	1.69 ± 0.67	0.76 ± 0.47	0
**Discards**		7.04 ± 1.34	6.55 ± 1.25	8.72 ± 1.79	8.20 ± 2.05	8.57 ± 1.53	1.33 ± 0.34
***Fish damaged***		4.60 ± 0.72 (70%)	4.65 ± 0.76 (79%)	6.12 ± 1.40 (71%)	6.39 ± 1.71 (80%)	4.57 ± 1.29 (53%)	1.14 ± 0.46 (86%)
***Commercial value***		0.21 ± 0.10 (3%)	0.48 ± 0.29 (8%)	0.09 ± 0.09(1%)	0.11 ± 0.11 (1%)	0.19 ± 0.19 (2%)	0
***Small size***		0.37 ± 0.17 (17%)	0.21 ± 0.10 (13%)	1.37 ± 0.72 (17%)	1.71 ± 1.18 (19%)	1.71 ± 0.87 (20%)	0
***Fish bycatch***		0.68 ± 0.20 (10%)**	0.05 ± 0.05 (1%)	0.97 ± 0.46 (11%)**	0	2.10 ± 0.92 (25%)	0.19 ± 0.19 (14%)

*** *p< 0*.*001*

** *p < 0*.*005*

* *p < 0*.*05*

Number of fish catches per 1000 hooks (mean ± SE) for the most common commercial species and those discarded during the trials of night setting (N = 18 paired sets), weighted lines (N = 11) and artificial baits (N = 5). In case of European hake *Merluccius merluccius* (target species), only not discarded individuals are considered: “a” refers to the number of individuals while “b” denotes the kilograms of fish. Discards refer to the fish that was returned to the sea because: 1) specimens were in bad condition (Fish damaged), 2) did not have commercial value, 3) were too small (Small size) or 4) were non-commercial or protected species (Fish bycatch). Percentages refer to the proportion of each reason of discarding.

The proportion of catch discarded was similar between the control and experimental settings of the mitigation measures tested. However, we found a significantly lower proportion of discards due to fish bycatch when longlines were set at night (χ^2^ = 7.97, *df* = 1, *p* < 0.005) and in lines with additional weights (χ^2^ = 8.13, *df* = 1, *p* < 0.005). In night setting trials, these catches were mostly registered in the control lines (N = 13 individuals, 39% common stingray *Dasyatis pastinaca*, 31% the ocean sunfish *Mola mola*, 15% swordfish *Xiphias gladius*, *8%* Blue shark *Prionace glauca* and 8% Atlantic bluefin tuna *Thunnus thynnus*), while only one ocean sunfish was bycaught during the settings at night. Conversely, in the weighted line trials, all fish bycatch occurred in the control lines (N = 12, 50% swordfish, 25% common stingray and 25% the ocean sunfish).

### Practical applicability

We detected some operational problems during the tori line and weighted line tests. In strong wind (> 17 knots) and cross wind conditions the tori line was displaced towards the fishing gear. Under these conditions, the captain refused to deploy the tori line alleging it could entangle with the longline and compromise the fishermen’s safety. Moreover, fishermen perceived an increase of entanglements between the branchlines and hooks during the setting operations when they use longlines with the additional weights.

### Longline sink rate

The sink rate of longlines to 10 m depth varied according to the line configuration considered (Table 4, *F*_2,51_ = 64.01, *p* < 0.005). Non-floated longlines sank at a significantly slower rate than the Floated (post-hoc Tukey’s test: *p* < 0.005, Floated 2; *p* <0.005). In addition, the sinking of hooks from the Floated 2 was significantly slower than the Floated 1 (post-hoc Tukey’s test: p < 0.005).

**Table 4:** Time and sink rate to 10-m depth and 10-m access window by longline configuration.

Longline type	Time (sec.)	Sink rate (m/s)	AW (m)	Setting speed (kts)	N
**Floated 1**	63.38 ± 1.29	0.16 ± 0.00	169.50 ± 4.12	5.26 ± 0.01	33
**Floated 2**	74.57 ± 3.43	0.14 ± 0.01	203.34 ± 14.24	5.28 ± 0.19	13
**Non-floated**	144 ± 7.49	0.07 ± 0.00	138.80 ± 15.01	1.85 ± 0.13	8

Time needed (seconds) to arrive to 10 m depth and sink rate (meters per second, mean ± SE) of the hooks for different longline types: “Piedra–bola” system (Floated 1 = longline type used by *Cona C*.*B* and Floated 2 = *Mar endins*) and the configuration without floats used by the small-scale vessel *Vigilant* (Non-floated). Seabird access window (AW) corresponds to the distance astern where the hooks are at 10 m depth. The data used were collected from hooks located at the middle section of the longline and between the float and the weight in the floated longlines and between weights in the non-floated longlines. The setting speed (mean ± SE) for each longline type is specified in knots (kts).

The distance astern at which longline hooks were within 10 m of the surface (AW, Table 4) differed between the longline types (F_2,48_ = 9.25, p < 0.005). Floated 2 lines showed the greatest extent of the access window (post-hoc Tukey’s test: Floated 1; *p* < 0.05, Non-floated; *p* <0.005). Conversely, we found that the seabird access to baited hooks was shortest in the Non-floated configuration. Nevertheless, the shorter distance in Non-floated was not significant when it was compared to the Floated 1.

**Fig 5:**
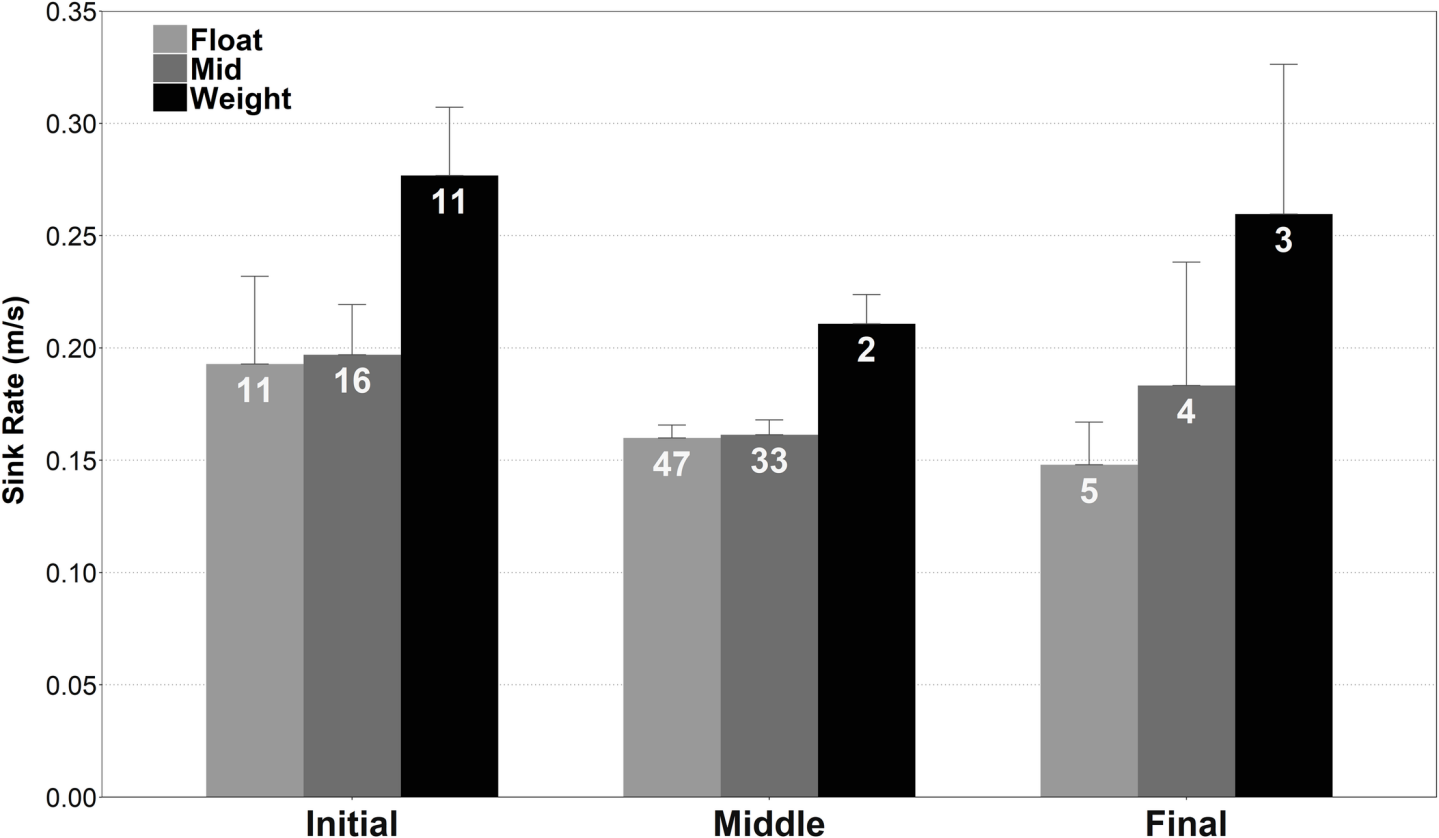
Sink rates of the baited hooks in the first 10 meters depth. The values (mean + CI 95%) came from the hooks located at (1) different positions inside of the float-weight sequence (Float = near to the float, Mid = between the float and the weight, Weight = near to the weight) and (2) longline sections (initial, middle and final). The hook sink rates were collected in the same vessel and from gear with a “Piedra–Bola” configuration (Floated 1). Numbers inside bars denote the sample sizes.

Hooks showed different sink rates depending on the longline section, and the position with respect to the weights and the floats (Fig 5). Hooks sank faster in the initial section (Mid_initial-middle_: *t*_17.728_ = 2.998, *p* < 0.05), and those near to the weight showed greater sink rates than those located near to the float or between the float and the weight (*F*_2,35_ = 8.98, *p* < 0.005; post-hoc Tukey’s test: Float_initial_−Weight_initial_, *p* < 0.005; Mid_initial_−Weight_initial_, *p* < 0.005). However, the proximity to the float did not reduce the sinking speed (Float_initial_—Mid_initial,_ ns; Float_middle_−Mid_middle_; ns). This could be due to the influence of the weight placed close to the float (see Table 1).

**Fig 6:**
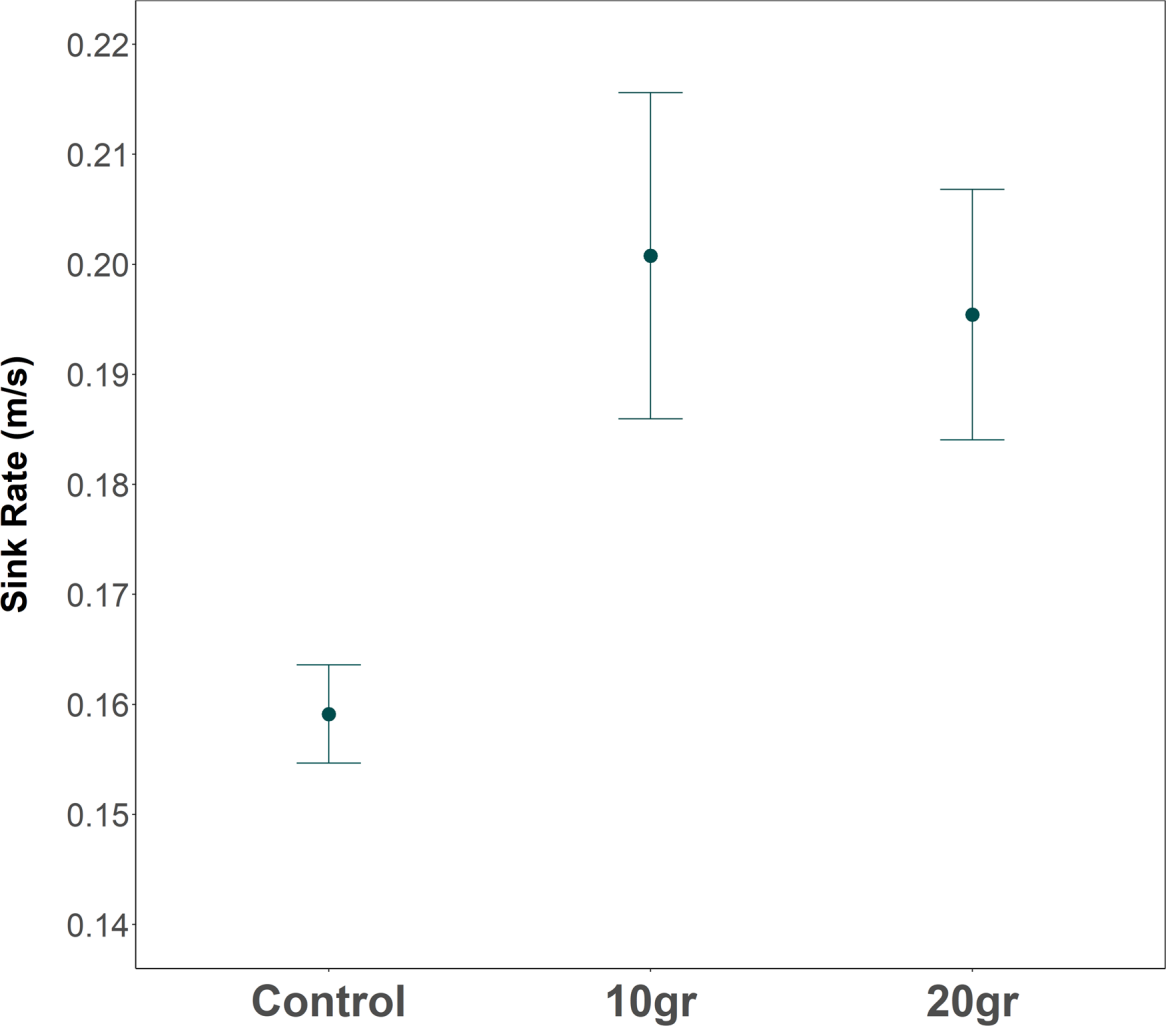
Sink-rate comparison of the longlines set with or without additional weights. The values (mean ± CI 95%) were registered in the control (n = 13) and experimental longlines with an added weight above hooks of 10 g (n = 14) and 20 g (n = 10). Data were collected from hooks located in the middle section of the longline and near to the floats.

The sinking speed of the hooks increased significantly when a lead weight of 10 g or 20 g was added to the branchlines (F_2,34_ = 15.83, p < 0.002, Fig 6), reaching the same sink rate for the two types of weights (10 g– 20 g = 0.20 m/s). Thus, in both cases, the addition of the weights led to a significant reduction of the seabird access window of at least 30 m (F_2,34_ = 22.02, p < 0.002; Mean ± SD; *Control* = 163.54 ± 7.27 m, *10 g* = 131.53 ± 17.96 m, *20 g* = 131.50 ± 14.31).

## Discussion

We assessed the efficiency and practical applicability of four mitigation measures on artisanal demersal longliners targeting European hake, the most valuable target species of this fleet in the western Mediterranean. Of the mitigation measures tested, night setting appears the most effective measure so far in minimizing seabird bycatch.

### Efficacy of mitigation tests on seabird interaction and fish catches

Night setting was the only method tested in the present study that reduced the frequency of bait attacks with no effect on target catches, and also had positive effect on fish bycatch species. In general, setting at night has proved to be very efficient in demersal longliners, especially in the Southern Ocean [34,35]. A recent study carried out in U.S. West Coast has also shown that night setting significantly reduces albatross catches without compromising target catches [36]. Nevertheless, its efficacy is particularly high among seabird species foraging only during daylight hours, but less effective on nocturnal seabirds. Some seabirds do forage at night, particularly during bright moonlight nights, which may compromise the efficacy of night setting [34,37,38]. In the Mediterranean, although all species are mainly diurnal, there are several species showing some nocturnal activity, such as Scopoli’s shearwater [32] and Audouin’s gull [33]. On-board observations conducted on demersal longliners in the same area showed the occurrence of nocturnal bycatch of these species and the Balearic shearwater in different moonlight conditions, but most of them occurred on full moon nights (71%, [18]). Even so, bird catches at night were far less frequent than catches at dawn or daytime. These findings were also found by Sánchez & Belda [39], which showed an important reduction of bait losses caused by Scopoli’s shearwater in longlines set at night. Consequently, both studies considered night setting as the most promising measure to reduce bycatch in the Mediterranean longliners. In the present study, we did not record any bycatch events during night settings, but we detected a large number of bait attacks by Scopoli’s shearwater in one of the night settings performed during a non-moon day, suggesting this measure may not completely avoid seabird catches and bycatch might not be restricted to moonlit nights. Furthermore, setting the longline at night may affect catches of commercial species, leading to economic losses for fishermen [35]. In this regard, this is the first study in the Mediterranean showing night setting having no effects on target catches. However, it increased the probability of catching the European conger, probably due to its nocturnal feeding behaviour [40]. This fact could lead to negative effects on fishermen activity if the night setting substantially increases the conger catches, since this species has low commercial value. Moreover, night setting reduced the catches of fish bycatch species, such as swordfish, ocean sunfish and blue shark. These species perform diel vertical migration so at night these fish stay close to the surface [41–43], making an encounter with demersal longline gear less likely. Nevertheless, these findings should be confirmed for longliners targeting fish other than European hake, since the effect of setting time may differ for other fish species with different activity rhythms [44]. Apart from reducing seabird catches, the potential benefits of night setting for fishermen is well illustrated by the fact that some fishermen operating near seabird breeding colonies voluntarily changed to setting their longlines at night to avoid bait losses caused by seabirds, suggesting benefits obtained by reducing bait losses compensate for any decrease in commercial catches or practical problems.

Tori lines have also been demonstrated to be a suitable mitigation method in demersal longliners [7]. However, its effectiveness may vary depending on the seabird assemblage and fishing gear configuration [7,24,36]. In this study, the deployment of the tori lines did not influence the overall number of bait attacks. Nonetheless, seabird attacks were displaced further astern, occurring beyond the 45 m covered by the streamers, and they were mainly performed by shearwaters. This can be explained by the greater diving ability of the different shearwater species compared to gull species which only feed on surface. Shearwaters can dive several metres and reach baited hooks at a considerable distance astern (mean ± SD; Scopoli’s shearwater: 0.78 ± 0.79 m [45], Balearic shearwater: 5.6 ± 4.1 m [46]). Indeed, *Puffinus* shearwaters can dive > 10 m depth (up to 28 m) [22], which means that according to the sinking rates recorded in the present study, shearwaters could still reach baited hooks after the 45 m covered by the streamers lines (when baited hooks are at 2 m depth) and up to 190 m behind the vessel (10 m depth). Therefore, the use of tori lines would appear to be ineffective in avoiding shearwater bycatch because baited hooks would still be accessible to these species. Previous studies also found that streamers were not able to reduce the catches of diving birds, only those of surface feeding birds [8,24,47]. Moreover, Gladics *et al*. [36] found that the tori line alone does not protect adequately the baits from bird attacks in the floated demersal longlines. The floats slow the sinking speed of the baited hooks, making them available to seabirds well beyond the area protected by the streamers lines, while also increasing the risk of fouling with the tori line. In addition, the effectiveness of the tori line may be compromised in low wind conditions, when birds attacked baits at shorter distances astern. This fact may also occur when fishermen reduce the vessel speed to change the box with the baited hooks. Shearwaters are the only group of seabirds bycaught during the trials, but all catches registered in tori line settings occurred under calm conditions. On windless days, the streamers did not move and did not deter birds away [48]. In addition, a lower turbulence of the water during calm days could favour the visibility of the baits to seabirds, and hence lead to an increase in bycatch risk [35]. Similar results were previously found by a relevant study [48], suggesting the efficacy of tori lines is limited to windy days. However, strong crosswinds may also compromise the effectiveness of the tori line by displacing it from the setting area, leading to entanglements with the gear, or by bringing it out of its ideal position to protect the baited hooks [7,24,49]. In fact, during these trials fishermen were concerned about the risk of tangles and refused to deploy the streamer line in strong winds (> 17 knots). To avoid the adverse influence of calm days and crosswinds, some studies recommended the use of paired scaring lines [7,24,50]. However, the installation of two tori lines on small artisanal demersal longliners would be impractical, compromising the fishing operations and increasing the chance of tangles [36].

Previous studies illustrated that weighted lines is the most suitable method to reduce catches of diving seabirds in demersal longliners, since it increases longline sink rate and reduces the seabird access window [7,51,52]. In addition, the increase in the longline sinking speed could also enhance the fishing efficiency, particularly for those vessels targeting demersal species of very deep waters, since the gears reaches the seafloor earlier, which maximises the bait attractiveness [53]. In these experiments, it was not possible to make a direct assessment of the effect of adding weights on bait attacks due to the low number of birds interacting with the vessel. Alternatively, to evaluate its effect on the bycatch risk we used the hook sink rates and its potential to prevent seabird access to baited hooks. We found that the lines with additional lead weights sank 25% faster than control lines, reducing by 20% (30 m) the seabird access. Even so, the sinking speed obtained (0.20 m/s) was not enough to reduce the catches of the shearwaters as they still had a large access window. Previous studies recommended achieving a longline sink rate of > 0.3 m/s to minimize seabird catches [54], but even though it does not entirely eliminated bycatch risk [7]. Consequently, some studies recommended the weighted line in combination with a tori line to achieve an effective reduction in the seabird catches, especially of diving species [7,24,50]. Regarding fish catches, weighted lines probably affected the way the line was settling on the seabed, since the lead weights could keep the branchlines in a more vertical position bringing the baited hooks closer to the seabed, ultimately affecting fish catches. Indeed, weighted lines increased blackmouth catshark catches, possibly because catsharks forages in the near bottom layer and on the seabed [55]. In addition, we found an interesting reduction of non-commercial or protected fish species (common stingray, swordfish and ocean sunfish) in experimental lines, which might also result from the different way the line arrangement on the seabed.

Artificial baits have been proposed as a potential method to reduce seabird bycatch in longline fisheries [3], however, there is little information about its effectiveness in reducing seabird bycatch and its effect on commercial catches. In this study, similar to the conditions of weighted lines, it was not possible to assess its effect on seabird interactions due to the low number of birds recorded during the control and experimental settings. However, artificial bait setting showed a 77% reduction of the target catches relative to control lines. This result could be explained by differences in the release rate of the chemical feeding stimulants between baits, since this attribute greatly determines catch efficiency [56]. Therefore, this method may constitute an extra cost for fishermen to buy the bait, overall rendering it inappropriate for the profitable exploitation of European hake in the study area. Nevertheless, the potential usefulness of other types of artificial bait cannot be completely dismissed.

### Effects of longline configuration on seabird access area

The seabird access area to baited hooks may vary between vessels according to the longline configuration used, the setting speed and the mainline location of the hooks. Regarding the longline configuration used, the distance between consecutive weights was an important factor in sink rate variation since the increase in distance between weights reduced the sink rate when we compared line configurations with similar weights and set at the same speed. In this regard, a previous study conducted in the Balearic Sea found that longline configurations with a greater distance between the weights have a higher seabird bycatch risk [18]. Therefore, our study provides evidence that this difference in the bycatch risk with regard to the distribution of the weights could be mainly driven by the variation in the sink rate and its related seabird access window to the baited hooks. Moreover, a previous study found higher attack rates in longlines with floats in comparison with non-floated longlines, since the use of floats slow the sink rate of the hooks and, hence, increasing the seabird access to baits [36]. Conversely, in this study, the longline type used in the small-scale vessel (Non-floated) sank significantly slower than the “Piedra–Bola” system (Floated), mainly due to the combination of lighter weights and the greater distance between them. Besides, in the case of the floated longlines considered in this study (zigzag structure), the placement of a heavy weight (2.3 kg) at a short distance of the float (7–11 m) could have also led to an increased sink rate in this type of configurations. However, these results cannot be extrapolate to other longlines configurations with floats used by the fleet (e.g. the pyramidal structures [18]), since the space between the weight and the float could be larger.

Setting speed also influenced sink rates but to a lesser extent [57]. The seabird access window in the Non-floated longline was significantly shorter than in the Floated 2 (139 m vs. 203 m) and similar to Floated 1, probably caused by its lower setting speed, thus indicating that lowering the setting speed of the vessels can decrease bycatch risk by reducing the seabird access window. However, setting speed also determines the final arrangement of the fishing gear on the seabed; faster speeds allow a tension of the mainline which keeps the longline straight and expanded along the sea bottom [57]. Vessel speed is sometimes adjusted to obtain an appropriate arrangement of the gear due to the speed and direction of the currents or other operational reasons, and so regulating setting speed to manage bycatch risk would thus be impractical. The mainline location of the hooks was also an influential factor on bait exposure, since we found differences in sinking rates between different sections of the mainline. The first section of the longline together with the hooks near to the weight sank much faster. As a result, the middle section and the hooks located near to the float and between the float and the weight were more exposed to seabird bait attacks. In contrast, a previous study [58] showed the opposite results, as they found a higher number of seabird catches in the first part of the longline, probably because the propeller turbulence slows the sinking speed of the hooks. In our study, however, the hooks located in the first section sank faster due to the influence of the anchor, since at the beginning of the setting the anchor has not yet reached the seabed so it pulls the longline towards the bottom at higher speed.

## Conclusions

Ideally, to ensure compliance of fishermen with the mitigation measures, the bycatch mitigation strategy must not only minimize seabird catches, but also needs to be cost-effective, practical, safe, accompanied with economic or social incentives, easy to implement, easy to manage and it needs to increase awareness on seabird by-catch and the involvement of fishermen [27,59,60]. This study mainly focused on the first three topics, namely efficacy in reducing seabird bycatch, effects on fish catches and practical applicability.

The three-endemic species of shearwaters are the most affected seabirds in demersal longliners of the western Mediterranean. These species show negative population trends mainly caused by mortality in commercial fisheries. Therefore, the mitigation strategy in the study area should focus on immediate actions to reduce catches of these species.

Night setting stands out as the most appropriate mitigation measure for demersal longlines in the Mediterranean so far, since it was the most efficient in reducing seabird attraction and bait attacks without compromising target species or having negative effects on fish bycatch species. However, setting the longlines at night does not completely avoid the seabird incidental catches, particularly in those species with some nocturnal activity such us the Scopoli's shearwater, and may increase the fish commercial catches of nocturnal species, such us European conger. However, this measure could be implemented at low economic costs and its compliance can be monitored and enforced by regulating the fishing schedules of longliners. Tori line was not able to prevent bait attacks of most vulnerable species to bycatch, since shearwaters could still access to baited hooks far beyond the distance protected by the streamers. Besides, it was particularly ineffective under calm wind conditions and impractical in strong crosswinds. The use of additional weights and gear configurations with reduced distance between weights (e.g. Floated 1) showed a higher hook sink rate, leading to a significant reduction of the seabird access window to baited hooks. However, in the present study, this reduction was relatively small and the addition of weights was not enough to reduce seabird catches and resulted in some operational problems. Nevertheless, the significant reduction of the seabird access window constitutes a good indicator for believing that introducing other changes in gear structures (e.g. Chilean method [26,52]) or the addition of weight in some specific longlines may eventually become an effective alternative in reducing seabird bycatch [26]. Nevertheless, gear-specific assessment is required to get optimal and efficient changes in the longline configurations.

## Supporting information

S1 Table Explanatory description of the trials conducted in the artisanal demersal longliners. (DOCX)

S2 Table Number of birds following the vessel of each seabird species in the control (C) and experimental (E) settings of the mitigation measures tested. (DOCX)

S3 Table Number of bait attacks of each seabird species in the control (C) and experimental (E) settings of the mitigation measures tested. (DOCX)

S4 Table Number of bait attacks recorded in each sample of the two-paired longlines (control and experimental) for the different mitigation measures tested. (DOCX)

S5 Table Number of fish caught of each species in the control (C) and experimental (E) settings of the night setting, weighted lines and artificial line trials. (DOCX)

S6 Table Number of hakes caught in each sample for two-paired longlines (control and experimental) for the night setting, weighted lines and artificial line trials. (DOCX)

S7 Table Number of hakes discarded in each sample for two-paired longlines (control and experimental) for the night setting, weighted lines and artificial line trials. (DOCX)

S8 Table Kilograms of hake caught in each sample for two-paired longlines (control and experimental) for the night setting, weighted lines and artificial line trials. (DOCX)

S9 Table Number of catsharks caught in each sample for two-paired longlines (control and experimental) for the night setting, weighted lines and artificial line trials. (DOCX)

S10 Table Number of congers caught in each sample for two-paired longlines (control and experimental) for the night setting, weighted lines and artificial line trials. (DOCX)
