# Supplementary material for: Seabird bycatch mitigation trials in artisanal demersal longliners of the Western Mediterranean
Source: PLoS One. 2018 May 9;13(5):e0196731. doi: 10.1371/journal.pone.0196731 (PMC5942821; doi:10.1371/journal.pone.0196731)
Supplement: S1 Table — (DOCX) [file pone.0196731.s001.docx]

**Seabird bycatch mitigation trials in artisanal demersal longliners of the Western Mediterranean**

Verónica Cortés and Jacob González-Solís

**Supporting Information**

**S1 Table. Explanatory description of the trials conducted in the artisanal demersal longliners.**

| **Trial** | **Period** | **Area** | **Methods** |
| --- | --- | --- | --- |
| **Night setting** | May 2013  June 2014 | *Vilanova i la Geltrú* | **20 samples**.  2 paired longlines of 1050 hooks were set closely, distributed parallel at similar depth.  Control line = sunrise or daytime setting  Experimental line = night setting (1 hour before the sunrise, Nautical dawn)  All moon phases were covered.  Depth: 254 – 260 m |
| **Weighted line** | July 2013  July 2014 | *Vilanova i la Geltrú* | **15 samples**.  2 paired longline types were set closely and continuously with a similar depth.  Control line = typical gear (2013: 1050 hooks, 2014: 1260 hooks)  Experimental line = branchlines with lead weights of 10 g or 20 g 5 cm above the hooks (2013: 1050 hooks, 2014: 840 hooks).  Depth: 260 – 463 m |
| **Tori line** | May 2013 | *Vilanova i la Geltrú* | **5 samples**.  1 single line of 2100 hooks, first half of the line was set without tori line (control) and the other half with tori line (experimental). Longlines were set during sunrise or daytime.  Depth: 241 – 260 m |
|  | May 2014 | *Llançà* | **7 samples**.  2 paired longlines were set closely and continuously during the sunrise, the last one had the tori line installed (experimental). Number of hooks was variable between samples but it ranged between 800 and 1000 hooks set.  Depth: 117 – 195 m |
| **Artificial baits** | June 2013 | *Vilanova i la Geltrú* | **5 samples**.  2 paired longline were set closely and continuously with a similar depth. One line was baited with conventional bait (control: 1050 hooks) and the other one with artificial baits (experimental: 1050 hooks). Line types were alternated between samples.  Depth: 243 – 391 m |
