# Supplementary material for: Seabird bycatch mitigation trials in artisanal demersal longliners of the Western Mediterranean
Source: PLoS One. 2018 May 9;13(5):e0196731. doi: 10.1371/journal.pone.0196731 (PMC5942821; doi:10.1371/journal.pone.0196731)
Supplement: S2 Table — (DOCX) [file pone.0196731.s002.docx]

**Seabird bycatch mitigation trials in artisanal demersal longliners of the Western Mediterranean**

Verónica Cortés and Jacob González-Solís

**Supporting Information**

**S2 Table. Number of birds following the vessel of each seabird species in the control (C) and experimental (E) settings of the mitigation measures tested.**

|  | **Night setting** | | | **Tori line** | | | **Weighted line** | | | **Artificial bait** | | |
| --- | --- | --- | --- | --- | --- | --- | --- | --- | --- | --- | --- | --- |
| **Species** | **C** | **E** | **Total** | **C** | **E** | **Total** | **C** | **E** | **Total** | **C** | **E** | **Total** |
| LARAUD | 69 | 14 | 83 | 29 | 13 | 42 | 7 | 2 | 9 | 10 | 12 | 22 |
| HYDPEL | 41 | 1 | 42 | 14 | 8 | 22 | 8 | 5 | 13 | 3 | 5 | 8 |
| CALDIO | 37 | 4 | 41 | 27 | 40 | 68 | 4 | 5 | 9 | 6 | 1 | 7 |
| PUFMAU | 7 | 0 | 7 | 2 | 6 | 8 | 2 | 5 | 7 | 1 | 0 | 1 |
| STESAN | 4 | 0 | 4 | 0 | 0 | 0 | 0 | 0 | 0 | 0 | 0 | 0 |
| LARMIC | 3 | 0 | 3 | 23 | 34 | 57 | 0 | 0 | 0 | 1 | 3 | 4 |
| MORBAS | 3 | 0 | 3 | 2 | 0 | 2 | 0 | 0 | 0 | 0 | 0 | 0 |
| STEHIR | 2 | 0 | 2 | 0 | 0 | 0 | 0 | 0 | 0 | 0 | 0 | 0 |
| PUFYEL | 1 | 0 | 1 | 5 | 7 | 12 | 0 | 0 | 0 | 0 | 0 | 0 |
| Puffinus spp. | 0 | 0 | 0 | 2 | 1 | 3 | 0 | 0 | 0 | 0 | 0 | 0 |
| STEPOM | 0 | 0 | 0 | 1 | 0 | 1 | 0 | 0 | 0 | 0 | 0 | 0 |
| CATSKU | 0 | 0 | 0 | 1 | 0 | 1 | 1 | 0 | 1 | 0 | 0 | 0 |
| LARMEL | 0 | 0 | 0 | 0 | 1 | 1 | 0 | 0 | 0 | 1 | 0 | 1 |
| **Total** | 167 | 19 | 186 | 106 | 110 | 216 | 22 | 17 | 39 | 22 | 21 | 43 |
| **Hooks number** | 21,000 | 21,000 | 42,000 | 11,391 | 12,107 | 23,498 | 17,850 | 13,650 | 31,500 | 6,300 | 6,300 | 12,600 |

LARAUD = *Larus audouinii*, HYDPEL = *Hydrobates pelagicus*, CALDIO = *Calonectris diomedea*, PUFMAU = *Puffinus mauretanicus*, STESAN = *Sterna sandvicensis*, LARMIC = *Larus michahellis*, MORBAS = *Morus bassanus*, STEHIR = *Sterna hirundo*, PUFYEL = *Puffinus yelkouan*, STEPOM = *Stercorarius pomarinus*, CATSKU = *Catharacta skua*, LARMEL = *Larus melanocephalus*
