# Supplementary material for: Seabird bycatch mitigation trials in artisanal demersal longliners of the Western Mediterranean
Source: PLoS One. 2018 May 9;13(5):e0196731. doi: 10.1371/journal.pone.0196731 (PMC5942821; doi:10.1371/journal.pone.0196731)
Supplement: S3 Table — (DOCX) [file pone.0196731.s003.docx]

**Seabird bycatch mitigation trials in artisanal demersal longliners of the Western Mediterranean**

Verónica Cortés and Jacob González-Solís

**Supporting Information**

**S3 Table. Number of bait attacks of each seabird species in the control (C) and experimental (E) settings of the mitigation measures tested.**

|  | **Night setting** | | | **Tori line** | | | **Weighted line** | | | **Artificial bait** | | |
| --- | --- | --- | --- | --- | --- | --- | --- | --- | --- | --- | --- | --- |
| **Species** | **C** | **E** | **Total** | **C** | **E** | **Total** | **C** | **E** | **Total** | **C** | **E** | **Total** |
| LARAUD | 55 | 0 | 55 | 8 | 0 | 8 | 0 | 1 | 1 | 1 | 0 | 1 |
| CALDIO | 154 | 33 | 187 | 59 | 151 | 210 | 4 | 0 | 4 | 28 | 3 | 31 |
| PUFMAU | 20 | 0 | 20 | 10 | 12 | 22 | 8 | 0 | 8 | 1 | 2 | 3 |
| STESAN | 1 | 0 | 1 | 0 | 0 | 0 | 0 | 0 | 0 | 0 | 0 | 0 |
| LARMIC | 0 | 0 | 0 | 4 | 8 | 12 | 0 | 0 | 0 | 0 | 0 | 0 |
| PUFYEL | 1 | 0 | 1 | 3 | 8 | 11 | 0 | 0 | 0 | 0 | 0 | 0 |
| **Total** | 231 | 33 | 264 | 85 | 179 | 263 | 12 | 1 | 13 | 30 | 5 | 35 |
| **Hooks number** | 21,000 | 21,000 | 42,000 | 11,391 | 12,107 | 23,498 | 17,850 | 13,650 | 31,500 | 5,250 | 5,250 | 10,500 |

LARAUD = *Larus audouinii*, CALDIO = *Calonectris diomedea*, PUFMAU = *Puffinus mauretanicus*, STESAN = *Sterna sandvicensis*, LARMIC = *Larus michahellis*, PUFYEL = *Puffinus yelkouan*
