# Supplementary material for: Seabird bycatch mitigation trials in artisanal demersal longliners of the Western Mediterranean
Source: PLoS One. 2018 May 9;13(5):e0196731. doi: 10.1371/journal.pone.0196731 (PMC5942821; doi:10.1371/journal.pone.0196731)
Supplement: S4 Table — (DOCX) [file pone.0196731.s004.docx]

**Seabird bycatch mitigation trials in artisanal demersal longliners of the Western Mediterranean**

Verónica Cortés and Jacob González-Solís

**Supporting Information**

**S4 Table. Number of bait attacks recorded in each sample of the two-paired longlines (control and experimental) for the different mitigation measures tested.**

|  | **Night setting** | | **Tori line** | | **Weighted lines** | | **Artificial baits** | |
| --- | --- | --- | --- | --- | --- | --- | --- | --- |
| **Sample** | **C** | **E** | **C** | **E** | **C** | **E** | **C** | **E** |
| **1** | 10 | 0 | 7 | 0 | 0 | 0 | 0 | 0 |
| **2** | 2 | 0 | 15 | 12 | 0 | 0 | 0 | 0 |
| **3** | 9 | 0 | 1 | 0 | 0 | 1 | 0 | 0 |
| **4** | 0 | 0 | 4 | 0 | 0 | 0 | 1 | 0 |
| **5** | 5 | 0 | 2 | 0 | 0 | 0 | 29 | 3 |
| **6** | 0 | 0 | 0 | 0 | 0 | 0 | - | - |
| **7** | 1 | 0 | 0 | 4 | 0 | 0 | - | - |
| **8** | 0 | 0 | - | - | 8 | 0 | - | - |
| **9** | 69 | 33 | 0 | 0 | 0 | 0 | - | - |
| **10** | 17 | 0 | - | - | 0 | 0 | - | - |
| **11** | 14 | 0 | 0 | 65 | 0 | 0 | - | - |
| **12** | 0 | 0 | 51 | 82 | 0 | 0 | - | - |
| **13** | 0 | 0 | 1 | 0 | 0 | 0 | - | - |
| **14** | 3 | 0 | - | - | 4 | 0 | - | - |
| **15** | 41 | 0 | 7 | 0 | 0 | 0 | - | - |
| **16** | 0 | 0 | - | - | - | - | - | - |
| **17** | 0 | 0 | - | - | - | - | - | - |
| **18** | 0 | 0 | - | - | - | - | - | - |
| **19** | 0 | 0 | - | - | - | - | - | - |
| **20** | 60 | 0 | - | - | - | - | - | - |
