# Supplementary material for: Seabird bycatch mitigation trials in artisanal demersal longliners of the Western Mediterranean
Source: PLoS One. 2018 May 9;13(5):e0196731. doi: 10.1371/journal.pone.0196731 (PMC5942821; doi:10.1371/journal.pone.0196731)
Supplement: S5 Table — (DOCX) [file pone.0196731.s005.docx]

**Seabird bycatch mitigation trials in artisanal demersal longliners of the Western Mediterranean**

Verónica Cortés and Jacob González-Solís

**Supporting Information**

**S5 Table. Number of fish caught of each species in the control (C) and experimental (E) settings of the night setting, weighted lines and artificial line trials.**

|  | **Night setting** | | | **Weighted lines** | | | **Artificial baits** | | |
| --- | --- | --- | --- | --- | --- | --- | --- | --- | --- |
| **Species** | **C** | **E** | **Total** | **C** | **E** | **Total** | **C** | **E** | **Total** |
| *Merluccius merluccius* | 750 | 713 | 1463 | 442 | 345 | 787 | 325 | 74 | 399 |
| *Galeus melastomus* | 206 | 218 | 424 | 450 | 729 | 1179 | 32 | 16 | 48 |
| *Conger conger* | 11 | 21 | 32 | 24 | 16 | 40 | 4 | 0 | 4 |
| *Micromesistius poutassou* | 7 | 4 | 11 | 2 | 1 | 3 | 7 | 0 | 7 |
| *Scyliorhinus canicula* | 7 | 8 | 15 | 1 | 4 | 5 | 1 | 0 | 1 |
| *Brama brama* | 5 | 0 | 5 | 2 | 1 | 3 | 0 | 0 | 0 |
| *Dasyatis pastinaca* | 5 | 0 | 5 | 3 | 0 | 3 | 1 | 0 | 1 |
| *Mola mola* | 4 | 1 | 5 | 3 | 0 | 3 | 9 | 1 | 10 |
| *Polyprion americanus* | 4 | 7 | 11 | 3 | 3 | 6 | 0 | 0 | 0 |
| *Ilex coindetii* | 2 | 2 | 4 | 3 | 0 | 3 | 0 | 0 | 0 |
| *Phycis blennoides* | 2 | 0 | 2 | 2 | 1 | 3 | 0 | 0 | 0 |
| *Pollachius virens* | 2 | 0 | 2 | 0 | 0 | 0 | 0 | 0 | 0 |
| *Xiphias gladius* | 2 | 0 | 2 | 6 | 0 | 6 | 0 | 0 | 0 |
| *Lepidotus caudatus* | 1 | 0 | 1 | 0 | 0 | 0 | 0 | 0 | 0 |
| *Prionace glauca* | 1 | 0 | 1 | 0 | 0 | 0 | 0 | 0 | 0 |
| *Thunnus thynnus* | 1 | 0 | 1 | 0 | 0 | 0 | 0 | 0 | 0 |
| *Etmopterus spinax* | 0 | 1 | 1 | 0 | 0 | 0 | 0 | 0 | 0 |
| *Trachurus trachurus* | 0 | 0 | 0 | 2 | 0 | 2 | 0 | 0 | 0 |
| *Helicolenus dactylopterus* | 0 | 0 | 0 | 1 | 0 | 1 | 1 | 0 | 1 |
| *Trichiurus lepturus* | 0 | 0 | 0 | 1 | 0 | 1 | 0 | 0 | 0 |
| *Scomber japonicus* | 0 | 0 | 0 | 0 | 2 | 2 | 0 | 0 | 0 |
| *Galeorhinus galeus* | 0 | 0 | 0 | 0 | 0 | 0 | 1 | 0 | 1 |
| **Total** | 1010 | 975 | 1985 | 945 | 1102 | 2048 | 380 | 91 | 472 |
| **Hooks number** | 18,900 | 18,900 | 37,800 | 13,020 | 9,030 | 22,050 | 5,250 | 5,250 | 10,500 |
