# Supplementary material for: Seabird bycatch mitigation trials in artisanal demersal longliners of the Western Mediterranean
Source: PLoS One. 2018 May 9;13(5):e0196731. doi: 10.1371/journal.pone.0196731 (PMC5942821; doi:10.1371/journal.pone.0196731)
Supplement: S6 Table — (DOCX) [file pone.0196731.s006.docx]

**Seabird bycatch mitigation trials in artisanal demersal longliners of the Western Mediterranean**

Verónica Cortés and Jacob González-Solís

**Supporting Information**

**S6 Table. Number of hakes caught in each sample for two-paired longlines (control and experimental) for the night setting, weighted lines and artificial line trials.**

|  | **Night setting** | | **Weighted lines** | | **Artificial baits** | |
| --- | --- | --- | --- | --- | --- | --- |
| **Sample** | **C** | **E** | **C** | **E** | **C** | **E** |
| **1** | 40 | 25 | 47 | 48 | 68 | 15 |
| **2** | - | - | - | - | 71 | 18 |
| **3** | 77 | 89 | 45 | 32 | 41 | 6 |
| **4** | 38 | 51 | 51 | 3 | 69 | 9 |
| **5** | 114 | 93 | 51 | 60 | 76 | 26 |
| **6** | 21 | 38 | 12 | 31 | - | - |
| **7** | 72 | 95 | 60 | 53 | - | - |
| **8** | 65 | 37 | 19 | 34 | - | - |
| **9** | 44 | 49 | - | - | - | - |
| **10** | 24 | 22 | 32 | 20 | - | - |
| **11** | 9 | 11 | 28 | 28 | - | - |
| **12** | 16 | 20 | 60 | 24 | - | - |
| **13** | 10 | 9 | - | - | - | - |
| **14** | 58 | 31 | 37 | 12 | - | - |
| **15** | - | - | - | - | - | - |
| **16** | 43 | 46 | - | - | - | - |
| **17** | 20 | 12 | - | - | - | - |
| **18** | 44 | 25 | - | - | - | - |
| **19** | 20 | 31 | - | - | - | - |
| **20** | 35 | 29 | - | - | - | - |
