# Supplementary material for: Seabird bycatch mitigation trials in artisanal demersal longliners of the Western Mediterranean
Source: PLoS One. 2018 May 9;13(5):e0196731. doi: 10.1371/journal.pone.0196731 (PMC5942821; doi:10.1371/journal.pone.0196731)
Supplement: S7 Table — (DOCX) [file pone.0196731.s007.docx]

**Seabird bycatch mitigation trials in artisanal demersal longliners of the Western Mediterranean**

Verónica Cortés and Jacob González-Solís

**Supporting Information**

**S7 Table. Number of hakes discarded in each sample for two-paired longlines (control and experimental) for the night setting, weighted lines and artificial line trials.**

|  | **Night setting** | | **Weighted lines** | | **Artificial baits** | |
| --- | --- | --- | --- | --- | --- | --- |
| **Sample** | **C** | **E** | **C** | **E** | **C** | **E** |
| **1** | 5 | 1 | 15 | 22 | 4 | 2 |
| **2** | - | - | - | - | 3 | 0 |
| **3** | 2 | 6 | 10 | 2 | 5 | 0 |
| **4** | 4 | 3 | 12 | 3 | 3 | 2 |
| **5** | 9 | 6 | 5 | 5 | 10 | 2 |
| **6** | 1 | 2 | 3 | 4 | - | - |
| **7** | 2 | 4 | 11 | 9 | - | - |
| **8** | 3 | 0 | 7 | 2 | - | - |
| **9** | 2 | 5 | - | - | - | - |
| **10** | 4 | 2 | 1 | 0 | - | - |
| **11** | 4 | 5 | 4 | 4 | - | - |
| **12** | 5 | 6 | 4 | 2 | - | - |
| **13** | 1 | 5 | - | - | - | - |
| **14** | 8 | 5 | 0 | 2 | - | - |
| **15** | - | - | - | - | - | - |
| **16** | 8 | 3 | - | - | - | - |
| **17** | 2 | 12 | - | - | - | - |
| **18** | 4 | 6 | - | - | - | - |
| **19** | 7 | 7 | - | - | - | - |
| **20** | 3 | 5 | - | - | - | - |
