# Supplementary material for: Seabird bycatch mitigation trials in artisanal demersal longliners of the Western Mediterranean
Source: PLoS One. 2018 May 9;13(5):e0196731. doi: 10.1371/journal.pone.0196731 (PMC5942821; doi:10.1371/journal.pone.0196731)
Supplement: S8 Table — (DOCX) [file pone.0196731.s008.docx]

**Seabird bycatch mitigation trials in artisanal demersal longliners of the Western Mediterranean**

Verónica Cortés and Jacob González-Solís

**Supporting Information**

**S8 Table. Kilograms of hake caught in each sample for two-paired longlines (control and experimental) for the night setting, weighted lines and artificial line trials.**

|  | **Night setting** | | **Weighted lines** | | **Artificial baits** | |
| --- | --- | --- | --- | --- | --- | --- |
| **Sample** | **C** | **E** | **C** | **E** | **C** | **E** |
| **1** | 26.64 | 19.53 | 18.82 | 22.64 | 70.08 | 9.11 |
| **2** | - | - | - | - | 27.96 | 9.40 |
| **3** | 43.48 | 51.86 | 25.79 | 18.04 | 21.35 | 4.14 |
| **4** | 20.02 | 26.78 | 23.96 | 31.03 | 30.63 | 4.06 |
| **5** | 58.50 | 53.18 | 26.24 | 12.63 | 36.33 | 13.58 |
| **6** | 10.58 | 18.04 | 3.80 | 20.78 | - | - |
| **7** | 39.08 | 47.84 | 24.74 | 22.88 | - | - |
| **8** | 34.44 | 18.00 | 5.31 | 12.32 | - | - |
| **9** | 21.38 | 23.32 | - | 10.57 | - | - |
| **10** | 12.03 | 13.47 | 18.03 | 11.65 | - | - |
| **11** | 3.44 | 2.88 | 9.35 | 15.25 | - | - |
| **12** | 5.61 | 8.11 | 27.85 | 15.85 | - | - |
| **13** | 7.08 | 1.44 | - | - | - | - |
| **14** | 36.56 | 14.40 | 23.58 | 8.75 | - | - |
| **15** | - | - | - | - | - | - |
| **16** | 21.37 | 17.84 | - | - | - | - |
| **17** | 11.21 | 6.51 | - | - | - | - |
| **18** | 25.54 | 11.95 | - | - | - | - |
| **19** | 8.14 | 15.92 | - | - | - | - |
| **20** | 15.45 | 10.27 | - | - | - | - |
