# Supplementary material for: Seabird bycatch mitigation trials in artisanal demersal longliners of the Western Mediterranean
Source: PLoS One. 2018 May 9;13(5):e0196731. doi: 10.1371/journal.pone.0196731 (PMC5942821; doi:10.1371/journal.pone.0196731)
Supplement: S9 Table — (DOCX) [file pone.0196731.s009.docx]

**Seabird bycatch mitigation trials in artisanal demersal longliners of the Western Mediterranean**

Verónica Cortés and Jacob González-Solís

**Supporting Information**

**S9 Table. Number of catsharks caught in each sample for two-paired longlines (control and experimental) for the night setting, weighted lines and artificial line trials.**

|  | **Night setting** | | **Weighted lines** | | **Artificial baits** | |
| --- | --- | --- | --- | --- | --- | --- |
| **Sample** | **C** | **E** | **C** | **E** | **C** | **E** |
| **1** | 4 | 14 | 125 | 45 | 30 | 16 |
| **2** | - | - | - | - | 0 | 0 |
| **3** | 0 | 0 | 50 | 57 | 0 | 0 |
| **4** | 0 | 0 | 64 | 22 | 1 | 0 |
| **5** | 0 | 2 | 7 | 3 | - | - |
| **6** | 1 | 0 | 45 | 110 | - | - |
| **7** | 0 | 0 | 39 | 46 | - | - |
| **8** | 5 | 1 | 50 | 93 | - | - |
| **9** | 1 | 0 | - | - | - | - |
| **10** | 2 | 0 | 27 | 110 | - | - |
| **11** | 0 | 3 | 8 | 38 | - | - |
| **12** | 0 | 3 | 24 | 121 | - | - |
| **13** | 0 | 0 | - | - | - | - |
| **14** | 27 | 19 | 11 | 84 | - | - |
| **15** | - | - | - | - | - | - |
| **16** | 18 | 26 | - | - | - | - |
| **17** | 15 | 30 | - | - | - | - |
| **18** | 43 | 47 | - | - | - | - |
| **19** | 89 | 72 | - | - | - | - |
| **20** | 1 | 1 | - | - | - | - |
