# Supplementary material for: Seabird bycatch mitigation trials in artisanal demersal longliners of the Western Mediterranean
Source: PLoS One. 2018 May 9;13(5):e0196731. doi: 10.1371/journal.pone.0196731 (PMC5942821; doi:10.1371/journal.pone.0196731)
Supplement: S10 Table — (DOCX) [file pone.0196731.s010.docx]

**Seabird bycatch mitigation trials in artisanal demersal longliners of the Western Mediterranean**

Verónica Cortés and Jacob González-Solís

**Supporting Information**

**S10 Table. Number of congers caught in each sample for two-paired longlines (control and experimental) for the night setting, weighted lines and artificial line trials.**

|  | **Night setting** | | **Weighted lines** | | **Artificial baits** | |
| --- | --- | --- | --- | --- | --- | --- |
| **Sample** | **C** | **E** | **C** | **E** | **C** | **E** |
| **1** | 1 | 2 | 0 | 1 | 2 | 0 |
| **2** | - | - | - | - | 0 | 0 |
| **3** | 0 | 0 | 1 | 0 | 2 | 0 |
| **4** | 0 | 1 | 1 | 1 | 0 | 0 |
| **5** | 0 | 0 | 1 | 0 | - | - |
| **6** | 0 | 0 | 6 | 5 | - | - |
| **7** | 0 | 0 | 0 | 1 | - | - |
| **8** | 1 | 1 | 0 | 2 | - | - |
| **9** | 1 | 1 | - | - | - | - |
| **10** | 2 | 4 | 2 | 0 | - | - |
| **11** | 1 | 1 | 2 | 0 | - | - |
| **12** | 0 | 1 | 7 | 1 | - | - |
| **13** | 0 | 2 | - | - | - | - |
| **14** | 2 | 4 | 4 | 5 | - | - |
| **15** | - | - | - | - | - | - |
| **16** | 0 | 1 | - | - | - | - |
| **17** | 0 | 1 | - | - | - | - |
| **18** | 0 | 1 | - | - | - | - |
| **19** | 0 | 0 | - | - | - | - |
| **20** | 3 | 1 | - | - | - | - |
